# Metabolic Syndrome: An Important Risk Factor for Parkinson's Disease

**DOI:** 10.1155/2014/729194

**Published:** 2014-05-14

**Authors:** Pei Zhang, Bo Tian

**Affiliations:** ^1^Department of Neurobiology, Tongji Medical School, Huazhong University of Science and Technology, 13 Hangkong Road, Wuhan, Hubei 430030, China; ^2^Key Laboratory of Neurological Diseases, Ministry of Education, Wuhan, Hubei 430030, China

## Abstract

Metabolic syndrome is becoming commoner due to a rise in obesity rates among adults. Generally speaking, a person with metabolic syndrome is twice as likely to develop cardiovascular disease and five times as likely to develop diabetes as someone without metabolic syndrome. Increasing oxidative stress in metabolic syndrome and Parkinson's disease is mentioned in the comprehensive articles; however, the system review about clear relation between metabolic syndrome and Parkinson's disease is deficient. In this review, we will focus on the analysis that the metabolic syndrome may be a risk factor for Parkinson's disease and the preventions that reduce the incident of Parkinson's disease by regulating the oxidative stress.

## 1. Introduction

Metabolic syndrome is a prevalent and increasing public health problem worldwide related to many chronic diseases. Its components mainly include at least insulin resistance, central obesity, glucose intolerance, dyslipidemia with elevated triglycerides, low HDL cholesterol, microalbuminuria, predominance of small dense LDL-cholesterol particles, hypertension, endothelial dysfunction, high waist circumference, oxidative stress, inflammation, tumors, neurodegeneration, and atherosclerosis-based ischemic cardio-  or cerebral-vascular disease. Meanwhile, recent studies have indicated that increased oxidative stress is the core and a general character of metabolism-related disease. Parkinson's disease, during the past decades, is one of the most frequent neurodegenerative disorders that cause dementia and it is one of the leading chronic diseases in all countries and it also displays the high level of reactive oxygen species (ROS). A growing body of evidence that has implicated the components of metabolic syndrome may contribute to the pathophysiology of Parkinson's disease. In the current brief review, we extend this work to search for findings from studies that provide evidence to clarify it and propose some prevention to delay the progression of Parkinson's disease via regulating the oxidative homeostasis.

## 2. The Components of Metabolic Syndrome Act as the Risk Factors for Parkinson's Disease

Risk factors for Parkinson's disease are either the result of genetic susceptibility (e.g., SNCA, PARK, PINK, and LRRK2 single nucleotide polymorphisms) or environmental exposure of a person's health to an event that can accelerate or further worsen dysfunction of the central nerve system. Metabolic syndrome is a crucial element of the environmental exposure of the global human health. Following up we will, respectively, introduce the components of metabolic syndrome that act as the risk factors for Parkinson's disease.

### 2.1. Fat and Obesity

Obesity continues to increase rapidly in the United States [1] and it is well established that obesity can increase the risk of Parkinson's disease and decrease life expectancy. A study has proved that high skinfold thickness in midlife was associated with Parkinson's disease [2]. And another study found that obesity in middle age increases the risk of future dementia independently of comorbid conditions. Perhaps adiposity works together with other risk factors to increase neurodegenerative disease [3]. In addition, some evidence shows that body mass index is associated with a risk of Parkinson's disease and the effect is graded and independent of other risk factors [4].

In an animal model of Parkinson's disease, high fat diet may lower the threshold for developing Parkinson's disease through affecting glucose transport and decreasing phosphorylation of HSP27 and degradation of I*κ*B*α* in the nigrostriatal system, at least following dopamine-specific toxin exposure [5, 6]. Moreover, increasing inflammatory signaling, adipokine levels, oxidative or nitrosative stress, mitochondrial dysfunction, and lipid metabolism have all been shown to occur with high fat feeding [7–9].

### 2.2. Glucose, Hyperglycemia, Insulin Resistance, and Diabetes

High glucose induced cell death is sustained by oxidative, nitrosative stress and mitochondrial superoxide generation through cleavage of the caspase 3 to regulate the apoptotic pathway [10–14]. In aging, hyperglycemia is also associated with Parkinson's disease through damage in central nervous system, a consequence of long-term exposure to glucose [15, 16]. Indeed, epidemiologic studies have implicated that prior type 2 diabetes is also the risk factor of developing Parkinson's disease [17]. Although, in different regions, the Parkinson's disease patients' brain exhibits similar cellular and functional changes with signs of increased oxidative stress, reduced mitochondrial function, reduced glucose uptake, and increased peroxidation of cellular membranes [18].

### 2.3. Hypertension

Many studies have been carried out on this topic: whether hypertension is the risk factor for Parkinson's disease. Much work, both theoretical and practical, has been reported recently in this field that hypertension is less frequent in Parkinson's disease patient than general population and others show that there is no difference between Parkinson's disease patients and healthy people [19, 20]. Nonetheless, a large prospective study suggested that Parkinson's disease risk is not significantly related to history of hypertension (RR = 0.96; 95% CI = 0.80 to 1.15) [21]. Although a lot of effort is being spent on proving the relation between Parkinson's disease and hypertension, the surely inerrable conclusion has yet to be reached.

### 2.4. Hyperhomocysteinemia and Endothelial Dysfunction

Hyperhomocysteinemia, a risk factor for endothelial dysfunction [22], has been involved in the pathophysiology of neurodegenerative disorders such as Alzheimer disease and Parkinson disease [23]. And homocysteine leads to endothelial dysfunction that hydrogen peroxide plays a critical role in mediating cell injury* in vitro* [24]. Large increases in cellular oxidative stress and inflammations occurred in response to high homocysteine that induced toxicity by decreased NAD+ [25–29]. In comparison, recent studies have also demonstrated that homocysteine is largely involved in antioxidant and reductive cellular biochemistry [30].

### 2.5. Inflammations

The involvement of inflammation in Parkinson's disease was initially proposed by McGeer et al. [31] who described the upregulation of HLA-DR-positive reactive microglia in the substantia nigra of Parkinson's disease patients in 1988. Additionally, they also reported that activated microglia was a contributor of proinflammatory and neurotoxic factors in Parkinson's disease patients [32]. Neuroinflammation which was induced by exposure to either toxicants or infectious agents with proinflammatory characteristics as a major factor in the pathogenesis of PD is wildly accepted at present. Plenty of cytokines such as tumor-necrosis factor-*α* (TNF-*α*) [32, 33], interleukin 1*β* (IL-1*β*) and IL-6 [32, 34–36], and the quantities of ROS [32] have been postulated to be involved in the etiology of Parkinson's disease. Furthermore, recent evidence indicates that endoplasmic reticulum (ER) stress [37–40] and inflammation coordinate the pathogenesis of Parkinson's diseases.

## 3. Targeting Oxidative Homeostasis as a Therapeutic Strategy against Parkinson's Disease

A growing number of studies have been completed to confirm that stimulation of oxidative stress that initiates apoptosis in many cells and animal models [11, 14, 41] is pivotal to the evolution of metabolic syndrome, diabetes, diabetic neuropathy, and several neurodegenerative disorders, such as Parkinson's disease and Alzheimer disease [42–46]. Though application of antioxidants and some measures in the field of preventing Parkinson's disease have proliferated in recent years, a phyletic classification is lacking. Here we introduce the potential mechanism under a variety of antioxidants or other therapeutic strategies to reduce the oxidation stress.

### 3.1. Plant Extract

Previous works, such as Bournival et al. [41, 47], Bureau et al. [48], and G$\overset{´}{\text{e}}$linas and Martinoli [49], reported that several plant extracts are powerful in neuroprotective activity of dopaminergic neurons against the oxidative burden provoked by administration of the potent parkinsonian toxin MPP+* in vitro* or 1-methyl-4-phenyl-1,2,3,6-tetrahydropyridine (MPTP)* in vivo*. The plant extract, which contains resveratrol and quercetin and sesamin [41, 47, 48], fermented papaya preparation [50], cinnamon polyphenols [51], and estradiol and phytoestrogens [49], was inhibited by oxidative stress that damages the normal physiological function of cellular organelle by regulating caspase 3, DNA fragment, estrogen receptors, cytokines, Akt, p38, MAPK, and ERK pathway.

An additional research which focuses on the extremely important antioxidant properties of cannabinoids, extract of hemp plant, may contribute to the neuroprotective effect in Parkinson's disease through banding the canonical cannabinoid CB1 and CB2 receptors [52–55].

### 3.2. Uric Acid

A large community-based survey indicated that the associated higher serum uric acid was able to decrease the prevalence of Parkinson's disease [56]. Similarly, it has been observed that UA levels in the serum of patients with Parkinson's disease are lower than in controls and that increased levels of UA are associated with a lower risk of Parkinson's disease [56–59]. Evidence was also proved that physiological concentration of uric acid would exert antioxidant effects, attenuating neuronal lesions caused by oxygen radicals, generated during an acute ischemic stroke and in cases of Parkinson's disease [60]. It had been established that the protective mechanisms of uric acid may be through regulating the DNA damage pathway [60–62]. The recent study from Massachusetts General Hospital found that the urate's ability to protect neurons requires the presence of astrocytes in Parkinson's disease unexpectedly [63].

### 3.3. Molecular Hydrogen

Hydrogen has great potential for improving oxidative stress-related diseases by inhaling H_2_ gas, injecting saline with dissolved H_2_, or drinking water with dissolved H_2_ [64]. Recent basic and clinical research has revealed that hydrogen is an important physiological regulatory factor with antioxidant, anti-inflammatory, and antiapoptotic protective effects on cells and organs [65]. Meanwhile, a large number of studies report that molecular hydrogen acts as a novel antioxidant and prevents or ameliorates diseases associated with oxidative stress in animal experiments [66–77] and clinical tests [78–81]. Molecular hydrogen improves obesity and diabetes by inducing hepatic FGF21 and stimulating fatty acid and glucose expenditure in mice [64]. Another research reported that molecular hydrogen is protective against 6-hydroxydopamine-induced nigrostriatal degeneration in a rat model of Parkinson's disease [75]. However, little is known about the mechanism that H_2_ acts on to prevent oxidative stress in Parkinson's disease.

### 3.4. Coffee and Caffeine Intake

Higher coffee and caffeine intake is associated with a significantly lower incidence of Parkinson's disease as discussed by Ross et al. [82]. Caffeine, a well-known central nervous system stimulant, inhibits the dopamine neurotransmission through adenosine receptor antagonism and mobilizes of intracellular calcium [83–85]. In addition, caffeine was regarded as an antioxidant against all the three reactive oxygen species, hydroxyl radical, peroxyl radical, and singlet oxygen [86].

### 3.5. Vitamin D and Vitamin E

Individuals with higher serum vitamin D concentrations showed a reduced risk of Parkinson disease. The relative risk between the highest and lowest quartiles was 0.33 (95% confidence interval, 0.14–0.80) [87]. Even so, the exact mechanisms by which vitamin D may protect against Parkinson disease are not fully understood [87]. High vitamin D status, however, has been shown to exhibit neuroprotective effects through antioxidative mechanisms, neuronal calcium regulation, immunomodulation, enhanced nerve conduction, and detoxification mechanisms [88–90]. Furthermore, the central issue in all these studies is to declare that high intake of dietary vitamin E [91, 92] may protect against the occurrence of PD, but vitamin C or *β* carotene does not [92]. And the protective influence for Parkinson's disease was seen with both moderate intake (relative risk: 0.81; 95% CI: 0.67–0.98) and high intake (0.78, 0.57–1.06) of vitamin E [92, 93].

### 3.6. Exercise

Inadequate physical activity has also been shown unequivocally to increase the morbidity and mortality rates of associated chronic disorders [94–96]. Exercise reduces the level of systemic inflammation by increasing the release of adrenaline, cortisol, growth hormone, prolactin, and other factors that have immunomodulatory effects and decreasing expression of toll-like receptors at the surface of monocytes, which have been suggested to be involved in mediating systemic inflammation [97–99]. Many results of the present research synthesis support the fact that the patients with PD improve their physical performance, activities of daily living [100, 101], and the effect of pharmacologic therapy [102] through exercise. The transcriptional coactivator PGC1*α* controls muscle plasticity and suppresses chronic systemic inflammation via repressing FOXO3 activity, increasing vascularization, ROS detoxification, and mitochondrial and metabolic gene expression [95]. The more specific mechanisms of the fact that exercise mediates the beneficial and advantageous effects for Parkinson's disease remain enigmatic.

## 4. Summary

**Figure 1:**
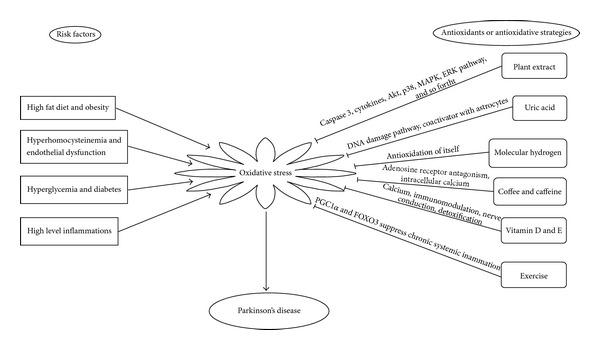
Summary of this review about oxidative stress and Parkinson's disease.

This review summarizes the data to support a link between oxidative stress and Parkinson's disease (Figure 1). Parkinson's disease (PD) is a progressive neurodegenerative disorder affecting the elder population mainly and its pathophysiology as well performs a metabolism-related dysfunction. It has been believed generally that oxidative stress was found during Parkinson's disease development when it occurs in early stage. Oxidative stress also is a crucial feather of metabolic syndrome. Undoubtedly, Parkinson's disease should be treated as a metabolic disease. Numbers of antioxidants are effective and efficient in the prevention and treatment of Parkinson's disease by modulating the oxidative stress, but Parkinson's disease whether or not is a metabolic syndrome still needs further epidemiological, basic science and clinical research. At present, considerable studies in a new direction are guiding future research on the relationship between Parkinson's disease and metabolic syndrome.

## References

[1] Mokdad AH, Serdula MK, Dietz WH, Bowman BA, Marks JS, Koplan JP (1999). The spread of the obesity epidemic in the United States, 1991–1998. *Journal of the American Medical Association*.

[2] Abbott RD, Ross GW, White LR (2002). Midlife adiposity and the future risk of Parkinson’s disease. *Neurology*.

[3] Whitmer RA, Gunderson EP, Barrett-Connor E, Quesenberry CP, Yaffe K (2005). Obesity in middle age and future risk of dementia: a 27 year longitudinal population based study. *British Medical Journal*.

[4] Hu G, Jousilahti P, Nissinen A, Antikainen R, Kivipelto M, Tuomilehto J (2006). Body mass index and the risk of Parkinson disease. *Neurology*.

[5] Morris JK, Bomhoff GL, Stanford JA, Geiger PC (2010). Neurodegeneration in an animal model of Parkinson’s disease is exacerbated by a high-fat diet. *American Journal of Physiology-Regulatory Integrative and Comparative Physiology*.

[6] Choi JY, Jang EH, Park CS, Kang JH (2005). Enhanced susceptibility to 1-methyl-4-phenyl-1,2,3,6-tetrahydropyridine neurotoxicity in high-fat diet-induced obesity. *Free Radical Biology and Medicine*.

[7] Cano P, Cardinali DP, Ríos-Lugo MJ, Fernández-Mateos MP, Reyes Toso CF, Esquifino AI (2009). Effect of a high-fat diet on 24-hour pattern of circulating adipocytokines in rats. *Obesity*.

[8] Gupte AA, Bomhoff GL, Swerdlow RH, Geiger PC (2009). Heat treatment improves glucose tolerance and prevents skeletal muscle insulin resistance in rats fed a high-fat diet. *Diabetes*.

[9] Uranga RM, Bruce-Keller AJ, Morrison CD (2010). Intersection between metabolic dysfunction, high fat diet consumption, and brain aging. *Journal of Neurochemistry*.

[10] Tsuruta R, Fujita M, Ono T (2010). Hyperglycemia enhances excessive superoxide anion radical generation, oxidative stress, early inflammation, and endothelial injury in forebrain ischemia/reperfusion rats. *Brain Research*.

[11] Obrosova IG, Drel VR, Pacher P (2005). Oxidative-nitrosative stress and poly(ADP-ribose) polymerase (PARP) activation in experimental diabetic neuropathy: the relation is revisited. *Diabetes*.

[12] Szabo C (2003). Multiple pathways of peroxynitrite cytotoxicity. *Toxicology Letters*.

[13] Vincent AM, Edwards JL, Sadidi M, Feldman EL (2008). The antioxidant response as a drug target in diabetic neuropathy. *Current Drug Targets*.

[14] Allen DA, Yaqoob MM, Harwood SM (2005). Mechanisms of high glucose-induced apoptosis and its relationship to diabetic complications. *Journal of Nutritional Biochemistry*.

[15] Tomlinson DR, Gardiner NJ (2008). Glucose neurotoxicity. *Nature Reviews Neuroscience*.

[16] Hu G, Jousilahti P, Bidel S, Antikainen R, Tuomilehto J (2007). Type 2 diabetes and the risk of Parkinson’s disease. *Diabetes Care*.

[17] Mercer LD, Kelly BL, Horne MK, Beart PM (2005). Dietary polyphenols protect dopamine neurons from oxidative insults and apoptosis: investigations in primary rat mesencephalic cultures. *Biochemical Pharmacology*.

[18] Pradhan AD, Manson JE, Rifai N, Buring JE, Ridker PM (2001). C-reactive protein, interleukin 6, and risk of developing type 2 diabetes mellitus. *Journal of the American Medical Association*.

[19] Scigliano G, Musicco M, Soliveri P, Piccolo I, Ronchetti G, Girotti F (2006). Reduced risk factors for vascular disorders in Parkinson disease patients: a case-control study. *Stroke*.

[20] Morano A, Jimenez-Jimenez FJ, Molina JA, Antolin MA (1994). Risk-factors for Parkinson’s disease: case-control study in the province of Caceres, Spain. *Acta Neurologica Scandinavica*.

[21] Simon KC, Chen H, Schwarzschild M, Ascherio A (2007). Hypertension, hypercholesterolemia, diabetes, and risk of Parkinson disease. *Neurology*.

[22] Woo KS, Chook P, Lolin YI (1997). Hyperhomocyst(e)inemia is a risk factor for arterial endothelial dysfunction in humans. *Circulation*.

[23] Kruman II, Culmsee C, Chan SL (2000). Homocysteine elicits a DNA damage response in neurons that promotes apoptosis and hypersensitivity to excitotoxicity. *Journal of Neuroscience*.

[24] Wall RT, Harlan JM, Harker LA, Striker GE (1980). Homocysteine-induced endothelial cell injury in vitro: a model for the study of vascular injury. *Thrombosis Research*.

[25] Gomes Trolin C, Regland B, Oreland L (1995). Decreased methionine adenosyltransferase activity in erythrocytes of patients with dementia disorders. *European Neuropsychopharmacology*.

[26] Blandini F, Fancellu R, Martignoni E (2001). Plasma homocysteine and L-DOPA metabolism in patients with Parkinson disease. *Clinical Chemistry*.

[27] Bottiglieri T, Hyland K (1994). S-adenosylmethionine levels in psychiatric and neurological disorders: a review. *Acta Neurologica Scandinavica, Supplementum*.

[28] Loscalzo J (1996). The oxidant stress of hyperhomocyst(e)inemia. *The Journal of Clinical Investigation*.

[29] Kurz K, Frick B, Fürhapter C (2013). Homocysteine metabolism in different human cells. *Issues*.

[30] Zappacosta B, Mordente A, Persichilli S (2001). Is homocysteine a pro-oxidant?. *Free Radical Research*.

[31] McGeer PL, Itagaki S, Boyes BE, McGeer EG (1988). Reactive microglia are positive for HLA-DR in the substantia nigra of Parkinson’s and Alzheimer’s disease brains. *Neurology*.

[32] Whitton PS (2007). Inflammation as a causative factor in the aetiology of Parkinson’s disease. *British Journal of Pharmacology*.

[33] Tansey MG, Frank-Cannon TC, McCoy MK (2008). Neuroinflammation in Parkinson’s disease: is there sufficient evidence for mechanism-based interventional therapy?. *Frontiers in Bioscience*.

[34] Mogi M, Harada M, Kondob T (1994). Interleukin-1*β*, interleukin-6, epidermal growth factor and transforming growth factor-*α* are elevated in the brain from parkinsonian patients. *Neuroscience Letters*.

[35] Blum-Degena D, Müller T, Kuhn W, Gerlach M, Przuntek H, Riederer P (1995). Interleukin-1*β* and interleukin-6 are elevated in the cerebrospinal fluid of Alzheimer’s and de novo Parkinson’s disease patients. *Neuroscience Letters*.

[36] Müller T, Blum-Degen D, Przuntek H, Kuhn W (1998). Interleukin-6 levels in cerebrospinal fluid inversely correlate to severity of Parkinson’s disease. *Acta Neurologica Scandinavica*.

[37] Imai Y, Soda M, Inoue H, Hattori N, Mizuno Y, Takahashi R (2001). An unfolded putative transmembrane polypeptide, which can lead to endoplasmic reticulum stress, is a substrate of Parkin. *Cell*.

[38] Imai Y, Soda M, Hatakeyama S (2002). CHIP is associated with Parkin, a gene responsible for familial Parkinson’s Disease, and enhances its ubiquitin ligase activity. *Molecular Cell*.

[39] Kaufman RJ (2002). Orchestrating the unfolded protein response in health and disease. *Journal of Clinical Investigation*.

[40] Imai Y, Soda M, Takahashi R (2000). Parkin suppresses unfolded protein stress-induced cell death through its E3 ubiquitin-protein ligase activity. *Journal of Biological Chemistry*.

[41] Bournival J, Quessy P, Martinoli M (2009). Protective effects of resveratrol and quercetin against MPP+ -induced oxidative stress act by modulating markers of apoptotic death in dopaminergic neurons. *Cellular and Molecular Neurobiology*.

[42] Whaley-Connell A, McCullough PA, Sowers JR (2011). The role of oxidative stress in the metabolic syndrome. *Reviews in Cardiovascular Medicine*.

[43] Zhou C, Huang Y, Przedborski S (2008). Oxidative stress in Parkinson’s disease: a mechanism of pathogenic and therapeutic significance. *Annals of the New York Academy of Sciences*.

[44] Vincent AM, Brownlee M, Russell JW (2002). Oxidative stress and programmed cell death in diabetic neuropathy. *Annals of the New York Academy of Sciences*.

[45] Rolo AP, Palmeira CM (2006). Diabetes and mitochondrial function: role of hyperglycemia and oxidative stress. *Toxicology and Applied Pharmacology*.

[46] Moreira PI, Santos MS, Seiça R, Oliveira CR (2007). Brain mitochondrial dysfunction as a link between Alzheimer’s disease and diabetes. *Journal of the Neurological Sciences*.

[47] Bournival J, Francoeur MA, Renaud J, Martinoli MG (2012). Quercetin and sesamin protect neuronal PC12 cells from high-glucose-induced oxidation, nitrosative stress, and apoptosis. *Rejuvenation Research*.

[48] Bureau G, Longpré F, Martinoli M (2008). Resveratrol and quercetin, two natural polyphenols, reduce apoptotic neuronal cell death induced by neuroinflammation. *Journal of Neuroscience Research*.

[49] Gélinas S, Martinoli M (2002). Neuroprotective effect of estradiol and phytoestrogens on MPP+-induced cytotoxicity in neuronal PC12 cells. *Journal of Neuroscience Research*.

[50] Aruoma OI, Hayashi Y, Marotta F, Mantello P, Rachmilewitz E, Montagnier L (2010). Applications and bioefficacy of the functional food supplement fermented papaya preparation. *Toxicology*.

[51] Cao H, Qin B, Panickar KS, Anderson RA (2008). Tea and cinnamon polyphenols improve the metabolic syndrome. *Agro Food Industry Hi-Tech*.

[52] García-Arencibia M, González S, de Lago E, Ramos JA, Mechoulam R, Fernández-Ruiz J (2007). Evaluation of the neuroprotective effect of cannabinoids in a rat model of Parkinson’s disease: importance of antioxidant and cannabinoid receptor-independent properties. *Brain Research*.

[53] Zuardi AW, Crippa JAS, Hallak JEC (2009). Cannabidiol for the treatment of psychosis in Parkinsons disease. *Journal of Psychopharmacology*.

[54] Booz GW (2011). Cannabidiol as an emergent therapeutic strategy for lessening the impact of inflammation on oxidative stress. *Free Radical Biology and Medicine*.

[55] Pacher P, Bátkai S, Kunos G (2006). The endocannabinoid system as an emerging target of pharmacotherapy. *Pharmacological Reviews*.

[56] Winquist A, Steenland K, Shankar A (2010). Higher serum uric acid associated with decreased Parkinson’s disease prevalence in a large community-based survey. *Movement Disorders*.

[57] Andreadou E, Nikolaou C, Gournaras F (2009). Serum uric acid levels in patients with Parkinson’s disease: their relationship to treatment and disease duration. *Clinical Neurology and Neurosurgery*.

[58] Álvarez-Lario B, Macarrón-vicente J (2011). Is there anything good in uric acid?. *QJM*.

[59] Chen H, Mosley TH, Alonso A, Huang X (2009). Plasma urate and Parkinson’s disease in the atherosclerosis risk in communities (ARIC) study. *American Journal of Epidemiology*.

[60] Cucuianu M, Brudasca I (2012). Gout, hyperuricemia and the metabolic syndrome. *Revista Româna de Medicina de Laborator*.

[61] Cutler RG (1991). Antioxidants and aging. *American Journal of Clinical Nutrition*.

[62] Ames BN, Cathcart R, Schwiers E, Hochstein P (1981). Uric acid provides an antioxidant defense in humans against oxidant- and radical-caused aging and cancer: a hypothesis. *Proceedings of the National Academy of Sciences of the United States of America*.

[63] Cipriani S, Desjardins CA, Burdett TC (2012). Urate and its transgenic depletion modulate neuronal vulnerability in a cellular model of Parkinson's disease. *PLoS ONE*.

[64] Kamimura N, Nishimaki K, Ohsawa I, Ohta S (2011). Molecular hydrogen improves obesity and diabetes by inducing hepatic FGF21 and stimulating energy metabolism in db/db mice. *Obesity*.

[65] Huang CS, Kawamura T, Toyoda Y, Nakao A (2010). Recent advances in hydrogen research as a therapeutic medical gas. *Free Radical Research*.

[66] Ohsawa I, Nishimaki K, Yamagata K, Ishikawa M, Ohta S (2008). Consumption of hydrogen water prevents atherosclerosis in apolipoprotein E knockout mice. *Biochemical and Biophysical Research Communications*.

[67] Fukuda K, Asoh S, Ishikawa M, Yamamoto Y, Ohsawa I, Ohta S (2007). Inhalation of hydrogen gas suppresses hepatic injury caused by ischemia/reperfusion through reducing oxidative stress. *Biochemical and Biophysical Research Communications*.

[68] Nakao A, Kaczorowski DJ, Wang Y (2010). Amelioration of rat cardiac cold ischemia/reperfusion injury with inhaled hydrogen or carbon monoxide, or both. *Journal of Heart and Lung Transplantation*.

[69] Buchholz BM, Kaczorowski DJ, Sugimoto R (2008). Hydrogen inhalation ameliorates oxidative stress in transplantation induced intestinal graft injury. *American Journal of Transplantation*.

[70] Hayashida K, Sano M, Ohsawa I (2008). Inhalation of hydrogen gas reduces infarct size in the rat model of myocardial ischemia-reperfusion injury. *Biochemical and Biophysical Research Communications*.

[71] Nagata K, Nakashima-Kamimura N, Mikami T, Ohsawa I, Ohta S (2009). Consumption of molecular hydrogen prevents the stress-induced impairments in hippocampus-dependent learning tasks during chronic physical restraint in mice. *Neuropsychopharmacology*.

[72] Nakashima-Kamimura N, Mori T, Ohsawa I, Asoh S, Ohta S (2009). Molecular hydrogen alleviates nephrotoxicity induced by an anti-cancer drug cisplatin without compromising anti-tumor activity in mice. *Cancer Chemotherapy and Pharmacology*.

[73] Cardinal JS, Zhan J, Wang Y (2010). Oral hydrogen water prevents chronic allograft nephropathy in rats. *Kidney International*.

[74] Fujita K, Seike T, Yutsudo N (2009). Hydrogen in drinking water reduces dopaminergic neuronal loss in the 1-methyl-4-phenyl-1,2,3,6-tetrahydropyridine mouse model of Parkinson’s disease. *PLoS ONE*.

[75] Fu Y, Ito M, Fujita Y (2009). Molecular hydrogen is protective against 6-hydroxydopamine-induced nigrostriatal degeneration in a rat model of Parkinson’s disease. *Neuroscience Letters*.

[76] Ohsawa I, Ishikawa M, Takahashi K (2007). Hydrogen acts as a therapeutic antioxidant by selectively reducing cytotoxic oxygen radicals. *Nature Medicine*.

[77] Oharazawa H, Igarashi T, Yokota T (2010). Protection of the retina by rapid diffusion of hydrogen: administration of hydrogen-loaded eye drops in retinal ischemia-reperfusion injury. *Investigative Ophthalmology and Visual Science*.

[78] Kajiyama S, Hasegawa G, Asano M (2008). Supplementation of hydrogen-rich water improves lipid and glucose metabolism in patients with type 2 diabetes or impaired glucose tolerance. *Nutrition Research*.

[79] Nakao A, Toyoda Y, Sharma P, Evans M, Guthrie N (2010). Effectiveness of hydrogen rich water on antioxidant status of subjects with potential metabolic syndrome-an open label pilot study. *Journal of Clinical Biochemistry and Nutrition*.

[80] Nakayama M, Kabayama S, Nakano H (2009). Biological effects of electrolyzed water in hemodialysis. *Nephron-Clinical Practice*.

[81] Suzuki Y, Sano M, Hayashida K, Ohsawa I, Ohta S, Fukuda K (2009). Are the effects of *α*-glucosidase inhibitors on cardiovascular events related to elevated levels of hydrogen gas in the gastrointestinal tract?. *FEBS Letters*.

[82] Ross GW, Abbott RD, Petrovitch H (2000). Association of coffee and caffeine intake with the risk of Parkinson disease. *Journal of the American Medical Association*.

[83] Nehlig A, Daval J-L, Debry G (1992). Caffeine and the central nervous system: mechanisms of action, biochemical, metabolic and psychostimulant effects. *Brain Research Reviews*.

[84] Popoli P, Caporali MG, Scotti de Carolis A (1991). Akinesia due to catecholamine depletion in mice is prevented by caffeine. Further evidence for an involvement of adenosinergic system in the control of motility. *Journal of Pharmacy and Pharmacology*.

[85] Daly JW (2007). Caffeine analogs: biomedical impact. *Cellular and Molecular Life Sciences*.

[86] Devasagayam TP, Kamat JP, Mohan H, Kesavan PC (1996). Caffeine as an antioxidant: inhibition of lipid peroxidation induced by reactive oxygen species. *Biochimica et Biophysica Acta*.

[87] Knekt P, Kilkkinen A, Rissanen H, Marniemi J, Sääksjärvi K, Heliövaara M (2010). Serum vitamin D and the risk of Parkinson disease. *Archives of Neurology*.

[88] Buell JS, Dawson-Hughes B (2008). Vitamin D and neurocognitive dysfunction: preventing “D”ecline?. *Molecular Aspects of Medicine*.

[89] Newmark HL, Newmark J (2007). Vitamin D and Parkinson’s disease—a hypothesis. *Movement Disorders*.

[90] Eyles DW, Smith S, Kinobe R, Hewison M, McGrath JJ (2005). Distribution of the Vitamin D receptor and 1*α*-hydroxylase in human brain. *Journal of Chemical Neuroanatomy*.

[91] de Rijk MC, Breteler MM, den Breeijen JH (1997). Dietary antioxidants and Parkinson disease. The Rotterdam study. *Archives of Neurology*.

[92] Etminan M, Gill SS, Samii A (2005). Intake of vitamin E, vitamin C, and carotenoids and the risk of Parkinson’s disease: a meta-analysis. *Lancet Neurology*.

[93] Miller ER, Pastor-Barriuso R, Dalal D, Riemersma RA, Appel LJ, Guallar E (2005). Meta-analysis: high-dosage vitamin E supplementation may increase all-cause mortality. *Annals of Internal Medicine*.

[94] Erikssen G, Liestøl K, Bjømholt J, Thaulow E, Sandvik L, Mrikssen J (1998). Changes in physical fitness and changes in mortality. *The Lancet*.

[95] Handschin C, Spiegelman BM (2008). The role of exercise and PGC1*α* in inflammation and chronic disease. *Nature*.

[96] Hu FB, Willett WC, Li T, Stampfer MJ, Colditz GA, Manson JE (2004). Adiposity as compared with physical activity in predicting mortality among women. *New England Journal of Medicine*.

[97] Nieman DC (2003). Current perspective on exercise immunology. *Current Sports Medicine Reports*.

[98] Gleeson M, McFarlin B, Flynn M (2006). Exercise and toll-like receptors. *Exercise Immunology Review*.

[99] Gleeson M (2007). Immune function in sport and exercise. *Journal of Applied Physiology*.

[100] Crizzle AM, Newhouse IJ (2006). Is physical exercise beneficial for persons with Parkinson’s disease?. *Clinical Journal of Sport Medicine*.

[101] Reuter I, Engelhardt M, Stecker K, Baas H (1999). Therapeutic value of exercise training in Parkinson’s disease. *Medicine and Science in Sports and Exercise*.

[102] Palmer SS, Mortimer JA, Webster DD (1986). Exercise therapy for Parkinson’s disease. *Archives of Physical Medicine and Rehabilitation*.

